# Sexual autonomy and self-reported sexually transmitted infections among women in sexual unions

**DOI:** 10.1186/s13690-022-00796-4

**Published:** 2022-01-26

**Authors:** Collins Adu, Aliu Mohammed, Eugene Budu, James Boadu Frimpong, Justice Kanor Tetteh, Bright Opoku Ahinkorah, Abdul-Aziz Seidu

**Affiliations:** 1 Department of Health Promotion, Education and Disability Studies, Kwame Nkrumah University of Science and Technology, Kumasi, Ghana; 2 Department of Health, Physical Education and Recreation, University of Cape Coast, Cape Coast, Ghana; 3 Department of Population and Health, University of Cape Coast, Cape Coast, Ghana; 4 School of public health Faculty of Health, University of Technology Sydney, Sydney, Australia; 5 Department of Estate Management, Faculty of Built and Natural Environment, Takoradi Technical University, Takoradi, Ghana; 6 Centre for Gender and Advocacy, Takoradi Technical University, Takoradi, Ghana; 7 College of Public Health, Medical and Veterinary Sciences, James Cook University, Townsville, Australia

**Keywords:** Public health, Sexual autonomy, STIs, Sub-Saharan Africa, Women

## Abstract

**Background:**

Sexually transmitted infections (STIs) are major public health challenges worldwide. Despite the importance of sexual autonomy in the prevention and control of sexual and reproductive health disorders such as STIs, there are limited studies on the possible relationship between women’s sexual autonomy and self-reported STIs, especially in sub-Saharan Africa (SSA). This study, therefore, examined the association between sexual autonomy and self-reported STIs among women in sexual unions in SSA.

**Methods:**

Data from the Demographic and Health Survey (DHS) of 31 countries in SSA conducted between 2010 and 2019 were analysed. A total of 234,310 women in sexual unions were included in the study. Data were analysed using binary logistic regression models and the results were presented as crude odds ratios (cORs) and adjusted odds ratios (aORs) at 95% confidence interval (CI).

**Results:**

The prevalence of self-reported STIs among women in sexual unions in SSA was 5.8%. Approximately 83.0% of the women surveyed had sexual autonomy. Women who had no sexual autonomy were less likely to have self-reported STIs (cOR=0.52, CI: 0.46-0.54), compared to those who had sexual autonomy. Additionally, higher odds of self-reported STIs were found among women aged 25-29, compared to those aged 15-19 (aOR= 1.21, CI: 1.09-1.35); those who reside in urban areas, compared to those who reside in rural areas (aOR= 1.51, CI: 1.37-1.66) and those who were cohabiting, compared to those who were married (aOR= 1.65, CI: 1.52-1.79). On the other hand, lower odds of self-reported STIs were found among women who were exposed to newspapers (aOR= 0.89, CI: 0.82-0.95), those whose partners had primary education (aOR= 0.84, CI: 0.78-0.91), those who were not exposed to radio (aOR= 0.84, CI: 0.79-0.89), and working women (aOR= 0.86, CI: 0.80-0.93).

**Conclusions:**

Findings from this study suggest that sexual autonomy is a significant predictor of self-reported STIs among women in sexual unions in SSA. Thus, instituting policies and programs that empower women and improve their levels of sexual autonomy may result in increased self-reporting of symptoms associated with STIs which subsequently help in minimising STI-related complications. Also, policies aimed at enhancing women’s sexual autonomy may reduce the burden of STIs in SSA, especially among women in sexual unions.

## Background

Sexually transmitted infections (STIs) remain a major public health challenge affecting many individuals across the globe [1, 2]. Chlamydia, syphilis, trichomoniasis, human papilloma virus (HPV), and gonorrhea are among the common STIs that confront individuals worldwide [3, 4]. Even though most STIs are curable, their detrimental and rippling effects on the health and wellbeing of individuals and their families are enormous [5]. Evidence shows that STIs may result in adverse conditions such as infertility, ectopic pregnancy, pelvic inflammatory disease, and loss of eye sight as well as increasing risk of contracting HIV [1, 6–9].

Contemporarily, the methods of prevention and treatment of STIs have seen more scientific and technological advancements, making them cheaper and more effective [5]. Nonetheless, STI prevalence continues to rise. For example, the World Health Organization (WHO) reported that new cases of STIs in 2012 stood at nearly 367 million worldwide [10]. The infections were pervasive in sub-Saharan Africa (SSA), Latin America and Asia, with SSA alone contributing to approximately 93 million cases of STIs annually [3]. This prompts urgent need for in-depth focus on this public health issue in the sub-region. One possible way of providing apt prevention and control interventions for people who have contracted STIs is by self-reporting incidence of STIs at the health facility for subsequent treatment [11–13]. Hence, studies that focus on self-reported STIs (SR-STIs) are valuable for formulating and strengthening public health policies and interventions.

Evidence show that sexual autonomy contributes significantly to the odds of contracting STIs [14, 15]. Women’s sexual autonomy is a woman’s ability to make informed decisions about her own sexual health such as abstaining from sexual intercourse, using condom and contraception or opting for abortion services [16, 17]. Women’s sexual autonomy is also closely linked to empowerment which is regarded as an essential measure of a society’s level of development [18–20]. Essentially, women who are sexually autonomous are to some extent shielded from unwanted pregnancies. Such women also often have lower odds of contracting STIs compared to women who are not sexually autonomous [15, 20]. However, in the case of SR-STIs, women with sexual autonomy often have higher levels of awareness regarding their sexual and reproductive health [6, 21] which often leads to increased likelihood of detecting and reporting symptoms of STIs compared to those without sexual autonomy [13].

Despite the health benefits of women’s sexual autonomy and its possible association with SR-STIs, little research has been done on this phenomenon in SSA. Meanwhile, the few studies conducted only focused on SR-STIs among men in SSA [22], men who have sex with men (MSM) and the aged [23, 24]. Therefore, this study sought to examine the prevalence of sexual autonomy and SR-STIs and the relationship between these variables among women in sexual unions in SSA. Findings of the study could help direct policies and interventions aimed at reducing the prevalence of STIs among women in sexual unions in SSA.

## Methods

### Data source

The study used data from 31 sub-Saharan African countries’ Demographic and Health Survey (DHS). Specifically, we used data from the women's recode (IR) files. The DHS is a nationally representative survey that is conducted in over 85 low-and middle-income countries globally. It focuses on essential maternal and child health markers such as sexual autonomy and SR-STIs [25]. The survey employs a two-stage stratified sampling technique, which makes the data nationally representative. The study by Aliaga and Ruilin [26] provides details of the sampling process. A total of 234,310 respondents who had complete information on all the variables of interest were included in our study. We relied on the Strengthening the Reporting of Observational Studies in Epidemiology (STROBE) statement in writing the manuscript [27]. The dataset is freely available for download at: https://dhsprogram.com/data/available-datasets.cfm.

### Variables studied

*Dependent variable*: SR-STIs was the dependent variable in this study. It was derived by asking if a woman had a STI in the last 12 months preceeding the survey. This was confirmed by responding “yes” to having any of the ensuing conditions: an STI, having abnormal genital discharge, experiencing a genital ulcer or sore, or having an STI symptom [36]. This has been adopted in several previous studies as a measure of SR-STIs [28–31].

*Explanatory variables*: The main explanatory variable was sexual autonomy. This variable was a composite variable derived from “respondent can refuse sex,” “respondent can ask partner to use condom,” and “wife is justified in asking the husband to use condom.” The response categories of these variables were: “Yes” and “No”. The ‘Yes’ responses were coded ‘1’ and the ‘No’ responses were coded ‘0’. An index was created with all the “Yes” and “No” answers with scores ranging from 0 to 3. The scores 0 and 1 were labelled as “No” and 2 to 3 were labelled as “Yes”. A dummy variable was created with ‘0’ score being women who did not have sexual autonomy and ‘1’ if women had sexual autonomy [21]. Other explanatory variables included in the study were age (years) (15-19, 20-24, 25-29, 30-34, 35-39, 40-44, 45-49), educational level (No education, Primary, Secondary, Higher) place of residence (Urban, Rural), wealth quintile (Poorest, Poorer, Middle, Richer, Richer), marital status (Married, Cohabiting), partners’ educational level (No education, Primary, Secondary, Higher), multiple sexual partner (No, Yes), exposure to newspaper (No, Yes), radio (No, Yes), and television (No, Yes), and occupation (Not working, Working). 

### Data analyses

**Fig. 1:**
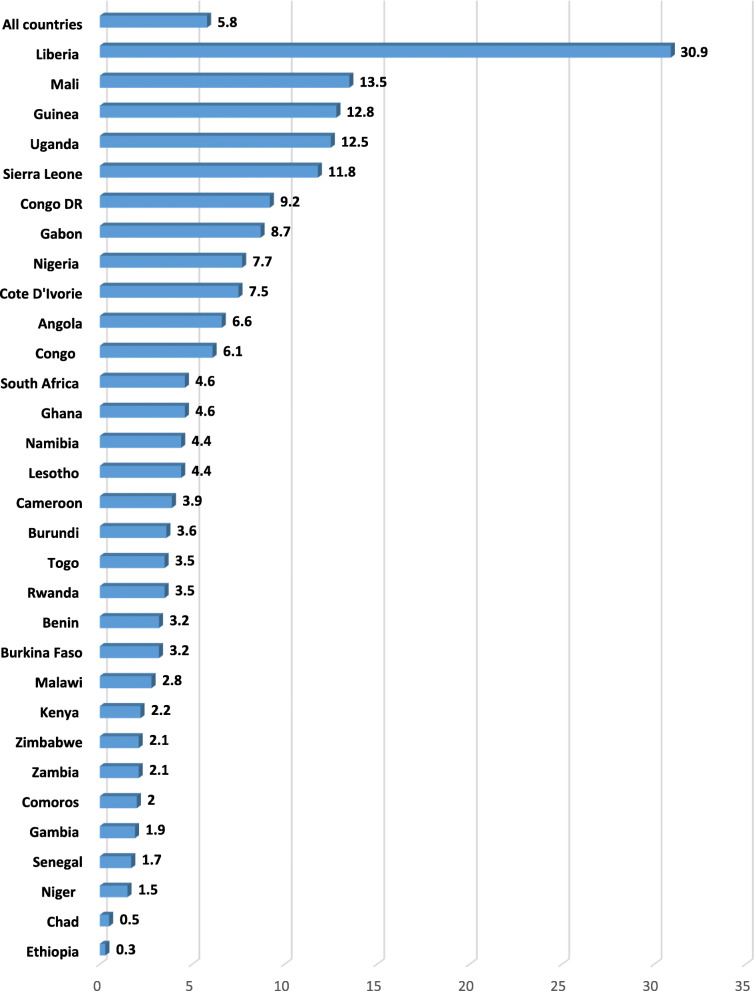
Prevalence of SR-STI among women in sexual union in SSA

**Fig. 2:**
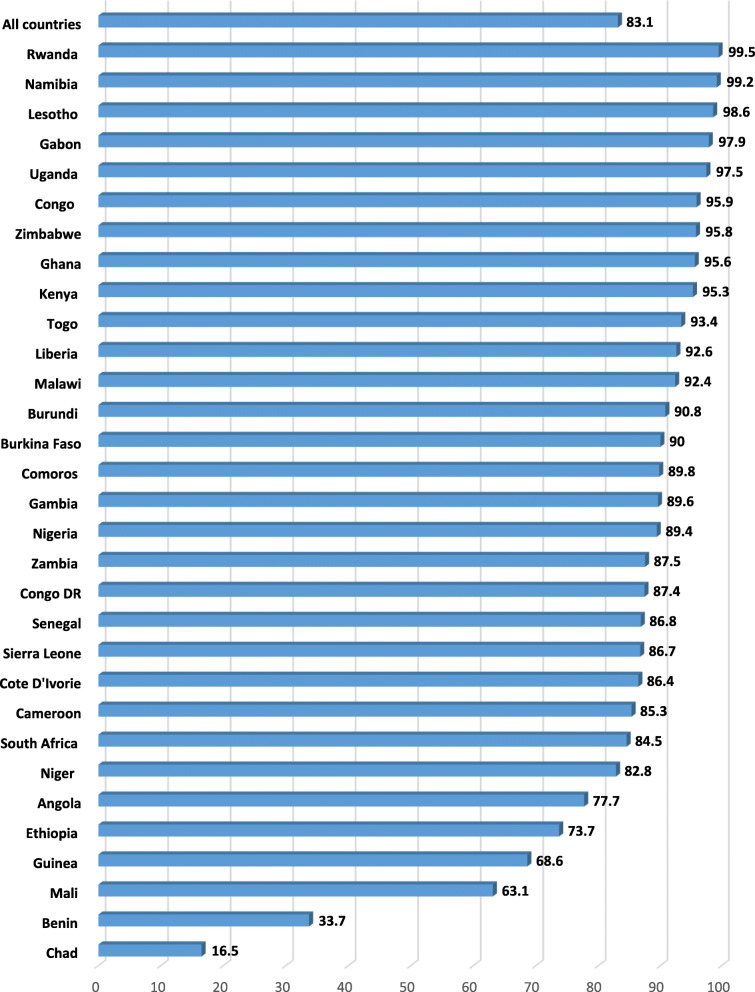
Prevalence of sexual autonomy among women in sexual union in SSA

Data was analysed with Stata version 16.0. The analysis was done in three steps. The first step was a graphical representation of the prevalence of SR-STIs  (Fig. 1) and sexual autonomy in SSA (Fig. 2) . The second step was a bivariate analysis that showed the proportion of SR-STIs across the explanatory variables with their p-values which were derived from a chi-square (Table 1). Variables that showed statistical significance from the Table 1 were moved to the third step of the analysis. In the third step of the analysis, two hierarchical logistic regression models were built. Model I looked at a bivariate analysis between the explanatory variables and SR-STIs. Model II controlled for the effect of all the covariates and country in a multivariable logistic regression. All frequency distributions were weighted while the survey command (svy) in Stata was used to adjust for the complex sampling structure of the data in the regression analyses.

**Table 1:** Description of study sample

Country	Year of survey	Weighted N	Weighted %
Angola	2015-16	6255	2.7
Burkina Faso	2010	12,993	5.5
Benin	2017-18	10,130	4.3
Burundi	2016-17	9493	4.0
Congo DR	2013-14	10,327	4.4
Congo	2011-12	5689	2.4
Cote D’Ivorie	2011-12	5296	2.3
Cameroon	2018	7435	3.2
Ethiopia	2016	8834	3.8
Gabon	2012	3785	1.6
Ghana	2014	4946	2.1
Gambia	2013	6112	2.6
Guinea	2018	5829	2.5
Kenya	2014	8086	3.4
Comoros	2012	2377	1.0
Liberia	2013	5050	2.2
Lesotho	2014	1687	0.7
Mali	2018	7555	3.2
Malawi	2015-16	15,501	6.6
Nigeria	2018	20,060	8.6
Niger	2012	7947	3.4
Namibia	2013	2771	1.2
Rwanda	2014-15	6755	2.9
Sierra Leone	2019	8821	3.8
Senegal	2010-11	8473	3.6
Chad	2014-15	10,137	4.3
Togo	2013-14	5545	2.4
Uganda	2016	10,494	4.5
South Africa	2016	2828	1.2
Zambia	2018	7233	3.1
Zimbabwe	2015	5866	2.5
Total		234,310	100

## Results

### Prevalence of SR-STIs and sexual autonomy among women in sexual union in SSA

Figure 1 presents results on the prevalence of self-reported STIs among women in sexual union in sub-Saharan African countries. On average, the prevalence of self-reported STIs was 5.8%. Women in Liberia had the highest prevalence (30.9%) while those from Ethiopia had the lowest (0.3%). In terms of the proportion of women who had sexual autonomy, prevalence of 83.1% was recorded in all the countries considered in this study. Women in Rwanda had the highest prevalence (99.5%) while those from Chad had the lowest (16.5%) (Fig. 2).

### Distribution of background characteristics and SR-STIs

We found a significant association between sexual autonomy and self-reported STIs among women in sexual union in SSA. Specifically, self-reported STIs was higher among women who had sexual autonomy (6.2%), compared to those who had no sexual autonomy (3.4%). There were significant variations in self-reported STIs across the socio-demographic characteristics of the women (marital status, multiple sexual partners, wealth quintile, exposure to radio, exposure to television, partner’s educational level, and place of residence) (Table 2).

**Table 2:** Background Characteristics and SR- STIs

Variables	Weighted N	Weighted %	Self-reported STI	p-value
**Sexual autonomy**				<0.0001
No	39,782	17.0	3.4	
Yes	194,528	83.0	6.2	
**Age (Years)**				<0.0001
15-19	14,507	6.2	4.8	
20-24	39,793	17.0	6.0	
25-29	50,961	21.7	6.4	
30-34	44,453	19.0	6.2	
35-39	37,588	16.0	5.9	
40-44	26,383	11.3	4.8	
45-49	20,624	8.8	4.4	
**Marital status**				<0.0001
Married	190,503	81.3	5.0	
Cohabiting	43,807	18.7	8.9	
**Occupation**				<0.0001
Working	63,177	27.0	5.0	
Not working	171,133	73.0	6.1	
**Multiple sexual partners**				0.0631
No	224,844	96.0	5.7	
Yes	9466	4.0	6.9	
**Wealth quintile**				<0.0001
Poorest	44,255	18.9	4.8	
Poorer	47,087	20.1	5.2	
Middle	46,876	20.0	5.7	
Richer	47,933	20.5	6.4	
Richest	48,158	20.5	6.6	
**Exposure to newspaper**				0.0001
No	196,418	83.8	5.6	
Yes	37,892	16.2	6.8	
**Exposure to radio**				<0.0001
No	96,508	41.2	5.0	
Yes	137,802	58.8	6.3	
**Exposure to television**				<0.0001
No	140,484	60.0	5.2	
Yes	96,826	40.0	6.6	
**Partner’s educational level**				<0.0001
No education	81,548	34.8	4.7	
Primary	61,457	26.2	5.2	
Secondary	71,144	30.4	7.2	
Higher	20,160	8.6	6.8	
**Place of residence**				<0.0001
Urban	81,726	34.9	7.5	
Rural	152,584	65.1	4.9	

^*^p<0.05, ^**^p<0.01, ^***^p<0.001

### Binary logistic regression analysis on the sexual autonomy and SR-STIs among women in sexual union in SSA

Table 3 shows results on the association between sexual autonomy and SR-STIs among women in sub-Saharan Africa. We found that compared to women who had sexual autonomy, those who had no sexual autonomy were less likely to have self-reported STIs (cOR=0.52, CI: 0.46-0.54) and this persisted after controlling for important covariates (aOR=0.57, CI: 0.52-0.64). In terms of the country-specific results, women who had sexual autonomy were less likely to have self-reported STIs in Lesotho (aOR= 0.02, CI: 0.01-0.16), Chad (aOR= 0.05, CI: 0.02-0.10), Benin (aOR= 0.15, CI: 0.11-0.19), Uganda (aOR= 0.42, CI: 0.24-0.72), Burkina Faso (aOR= 0.51, CI: 0.32-0.79), Guinea (aOR= 0.67, CI: 0.55-0.81), and Nigeria (aOR= 0.75, CI: 0.62-0.91) (see Model II of Table 4).

**Table 3:** Sexual autonomy and self-reported STIS among women in SSA

Country	Model I	Model II
**Sexual autonomy**	cOR(95%CI)	aOR(95%CI)
No	0.52^***^ (0.46-0.54)	0.57^***^ (0.52-0.64)
Yes	Reference (1.0)	Reference (1.0)
**Age**		
15-19	Reference (1.0)	Reference (1.0)
20-24	1.24^***^ (1.011-1.38)	1.15^*^ (1.03-1.29)
25-29	1.30^***^ (1.17-1.45)	1.21^***^ (1.09-1.35)
30-34	1.26^***^ (1.13-1.40)	1.19^**^ (1.07-1.32)
35-39	1.20^**^ (1.08-1.34)	1.14^*^ (1.02-1.28)
40-44	0.96 (0.81-1.08)	0.92 (0.82-1.04)
45-49	0.88^*^ (0.77-1.00)	0.87^*^ (0.76-0.99)
**Marital status**		
Married	Reference (1.0)	Reference (1.0)
Cohabiting	1.81^***^ (1.66-1.97)	1.65^***^ (1.52-1.79)
**Occupation**		
Working	0.84^***^ (0.78-0.91)	0.86^***^ (0.80-0.93)
Not working	Reference (1.0)	Reference (1.0)
**Wealth quintile**		
Poorest	0.73^***^ (0.66-0.80)	1.14^*^ (1.02-1.28)
Poorer	0.77^***^ (0.70-0.85)	1.16^**^ (1.04-1.30)
Middle	0.86^***^ (0.78-0.94)	1.18^**^ (1.06-1.31)
Richer	0.96 (0.88-1.05)	1.13^*^ (1.03-1.25)
Richest	Reference (1.0)	Reference (1.0)
**Exposure to newspaper**		
No	Reference (1.0)	Reference (1.0)
Yes	1.18^***^ (1.09-1.27)	0.89^***^ (0.82-0.95)
**Exposure to radio**		
No	0.74^***^ (0.70-0.79)	0.84^***^ (0.79-0.89)
Yes	Reference (1.0)	Reference (1.0)
**Exposure to television**		
No	Reference (1.0)	Reference (1.0)
Yes	1.34^***^ (0.26-1.43)	1.02 (0.94-1.10)
**Partner’s educational level**		
No education	Reference (1.0)	Reference (1.0)
Primary	1.02 (0.94-1.10)	0.84^***^ (0.78-0.91)
Secondary	1.44^***^ (1.32-1.56)	1.05 (0.87-1.11)
Higher	1.37^***^ (1.23-1.52)	0.99 (0.87-1.11)
**Place of residence**		
Urban	1.60^***^ (1.48-1.74)	1.51^***^ (1.37-1.66)
Rural	Reference (1.0)	Reference (1.0)

Exponentiated coefficients; 95% confidence intervals in brackets; aOR adjusted Odds Ratios; cOR: crude Odds Ratios; CI Confidence Interval

^*^p<0.05, ^**^p<0.01, ^***^p<0.001

**Table 4:** Sexual autonomy and SR-STIs in respective countries in SSA

Country	Model I cOR (95%CI)	Model II aOR (95%CI)
Angola	0.47^***^ (0.35-0.62)	0.77 (0.57-1.04)
Burkina Faso	0.42^***^ (0.27-0.66)	0.51^**^ (0.32-0.79)
Benin	0.15^***^ (0.12-0.20)	0.15^***^ (0.11-0.19)
Burundi	0.88 (0.58-1.32)	1.01 (0.67-1.52)
Congo DR	0.84 (0.69-1.01)	1.00 (0.82-1.21)
Congo	1.17 (0.77-1.77)	1.34 (0.87-2.06)
Cote D’Ivorie	0.87 (0.65-1.17)	0.98 (0.72-1.33)
Cameroon	0.45^***^ (0.29-0.70)	0.60^**^ (0.38-0.94)
Ethiopia	0.66 (0.34-1.27)	0.80 (0.40-1.61)
Gabon	1.28 (0.64-2.57)	1.50 (0.73-3.05)
Ghana	1.26 (0.73-2.15)	1.31 (0.76-2.28)
Gambia	0.74 (0.41-1.31)	0.83 (0.46-1.01)
Guinea	0.63^***^ (0.52-0.75)	0.67^***^ (0.55-0.81)
Kenya	0.36^**^ (0.16-0.80)	0.39^*^ (0.17-0.92)
Comoros	1.48 (0.70-3.16)	1.37 (0.62-3.03)
Liberia	0.99 (0.80-1.21)	1.13 (0.91-1.40)
Lesotho	0.05^***^ (0.03-0.06)	0.02^***^ (0.01-0.16)
Mali	0.97 (0.84-1.11)	1.16^*^ (1.00-1.34)
Malawi	0.67 (0.43-1.04)	0.65^*^ (0.42-1.02)
Nigeria	0.75^**^ (0.63-0.91)	0.75^***^ (0.62-0.91)
Niger	0.78 (0.47-1.31)	0.93 (0.55-1.57)
Namibia	1.29 (0.40-4.18)	1.56 (0.47-5.19)
Rwanda	1.47 (0.35-6.11)	1.74 (0.41-7.35)
Sierra Leone	0.38^***^ (0.30-0.50)	0.45^***^ (0.35-0.59)
Senegal	0.54^*^ (0.30-0.95)	0.66 (0.37-1.18)
Chad	0.04^***^ (0.02-0.89)	0.05^***^ (0.02-0.10)
Togo	0.52 (0.26-1.02)	0.65 (0.32-1.30)
Uganda	0.37^***^ (0.22-0.64)	0.42^***^ (0.24-0.72)
South Africa	0.58^*^ (0.31-1.02)	0.54^*^ (0.29-0.99)
Zambia	0.67 (0.39-1.15)	0.67 (0.39-1.15)
Zimbabwe	0.33 (0.08-1.35)	0.33 (0.08-1.34)

Exponentiated coefficients; 95% confidence intervals in brackets; aOR adjusted Odds Ratios; cOR: crude Odds Ratios; CI Confidence Interval

^*^p<0.05, ^**^p<0.01, ^***^p<0.001

Higher odds of SR-STIs were found among women aged 25-29 (aOR= 1.21, CI: 1.09-1.35), compared to those aged 15-19; those who reside in urban areas (aOR= 1.51, CI: 1.37-1.66) compared to those who reside in rural areas and those who were cohabiting (aOR= 1.65, CI: 1.52-1.79) compared to those who were married (see Model II of Table 3). Lower odds of self-reported STIs were found among women who were exposed to newspapers (aOR= 0.89, CI: 0.82-0.95), those whose partners had primary education (aOR= 0.84, CI: 0.78-0.91), those who were not exposed to radio (aOR= 0.84, CI: 0.79-0.89), and working women (aOR= 0.86, CI: 0.80-0.93) (see Model II of Table 3).

## Discussion

This study examined the association between sexual autonomy and SR-STIs among women in sexual unions using data from DHS of 31 countries in SSA. The findings revealed that the prevalence of SR-STIs among the women was 5.8%, and 83.1% of the women surveyed had sexual autonomy. Also, the study showed that there was significant association between sexual autonomy and SR-STIs among women in sexual unions in SSA. Specifically, women who had sexual autonomy were more likely to report STIs compared to those who had no sexual autonomy. This finding persisted even after controlling for marital status, multiple sexual partners, wealth quintile, exposure to radio, exposure to television, partner’s educational level and place of residence.

In this study, the overall prevalence of SR-STIs among women in sexual unions in SSA was 5.8%, and it ranged from 0.3% in Ethiopia to 30.9% in Liberia. Similar findings were reported in previous studies in SSA albeit at the individual country level [32, 33]. A recent multi-country study by Seidu et al. [22] reported similar but slightly lower prevalence of self-reported STIs (3.8%) among sexually active men in SSA. Perhaps, the higher prevalence figure recorded in the present study (5.8%) vis-à-vis the study by Seidu et al. [22] underscores the vulnerability of women to STIs relative to men [34]. This calls for increased attention and interventions towards addressing the issue of STIs among women in sexual unions in SSA. Meanwhile, the overall prevalence of STIs reported in the present study (5.8%) is lower than the 19.4% reported by WHO [35]. The variations in prevalence could be attributed to the differences in time frames for the studies and methods used in data collection. Whereas the WHO’s study relied on clinically confirmed incidence data on four curable STIs (Chlamydia, syphilis, trichomonas and gonorrhea) to determine prevalence, the present study used self-reported data on symptoms of STIs which include having abnormal genital discharge, experiencing a genital ulcer, or having an STI symptom [36]. Meanwhile, many STIs among women are asymptomatic [6] which might have accounted for the low prevalence rate in this study. This calls for increased use of laboratory based or diagnostic studies in determining the prevalence of STIs.

Also, the study showed that women who had sexual autonomy were more likely to have SR-STIs compared to those who had no sexual autonomy. Similar associations between sexual autonomy and SR-STIs was found by Nankinga et al. [33]. Available evidence suggests that women with sexual autonomy have higher levels of awareness and decision-making capacity regarding their sexual and reproductive health [6, 21]. This may increase their likelihood to detect and report symptoms of STIs compared to those without sexual autonomy [13]. Thus, we speculate that the high prevalence of SR-STIs among women with sexual autonomy is perhaps a function of their assertiveness and willingness to talk about their sexual health which include reporting STIs. Therefore, contrary to the claims by Nankinga et al. [33], we argue that the high prevalence of SR STIs among women with sexual autonomy may not necessarily be indicative of higher incidence of STIs relative to those without sexual autonomy. As suggested by Chesson et al. [6] low levels of sexual health awareness as well as stigma associated with reporting of genital symptoms often curtail reporting or delay healthcare seeking for STIs among women. Thus, increasing women’s level of sexual autonomy even if not protective against STIs, may increase the odds of early detection and reporting of STI-related symptoms, thereby minimizing complications associated with STIs among women. However, further laboratory-based studies are needed to ascertain whether women with sexual autonomy have higher incidence of STIs relative to those without sexual autonomy.

### Practical implications

In line with WHO’s Global Health Sector Strategy on STIs 2016-2021[34], this study provides important data on STI burden in SSA, especially among women in sexual unions [34]. The multi-country nature of the prevalence estimates improves our understanding of the burden of self-reported STIs in SSA. Additionally, our findings on the association between sexual autonomy and SR-STIs is important in designing and implementing strategies aimed at reducing burden of STIs in SSA. For instance, increasing the levels of sexual autonomy among women could result in increased self-reporting and early initiation of treatment [11, 12]. This could minimise STI-related complications such as ectopic pregnancy, infertility, pelvic inflammatory disease and chronic abdominal pain among the women [6]. Even though we speculate that high prevalence of SR-STIs among sexually autonomous women is probably due to their increased willingness to report and seek treatment compared to women without sexual autonomy, further studies are needed to ascertain this assertion.

## Strengths and limitations

The major strength of this study is the use of the most recent nationally representative cross-sectional datasets of 31 countries in SSA to examine the association between sexual autonomy and SR-STIs among women in sexual unions. Additionally, the rigorous data collection approach and analysis technique used in the present study enhances the generalisability of our findings to other women in sexual unions in SSA. Despite these strengths, the study has some limitations which need to be acknowledged. First of all, due to the use of cross-sectional study design, only associations between sexual autonomy and self-reported STIs were adduced but not causality. Also, the DHS data does not indicate the exact type of STI among respondents which limit the interpretation of our findings. Furthermore, the prevalence of STIs was limited to self-report and not medically diagnosed or laboratory confirmed which could limit the interpretation of the prevalence of STI among the women. Finally, there is a possibility of underreporting of STIs since some of the women might give socially desirable answers which could create biases in the study findings.

## Conclusions

Findings from this study suggest that sexual autonomy is a significant predictor of SR-STIs among women in sexual unions in SSA. Thus, instituting policies and programs that empower women to improve their levels of sexual autonomy may result in increased self-reporting of symptoms associated with STIs which could subsequently help in minimising STI-related complications. Also, policies aimed at enhancing women’s sexual autonomy may reduce the burden of STIs in SSA, especially among women in sexual unions.

## Data Availability

The datasets used and/or analyzed in the current study are available from the corresponding author on reasonable request. Besides this, since we used data from Demographic and Health Survey which is publicly available for research.

## Abbreviations

SSA: sub-Saharan Africa
STIs: sexually transmitted infections
DHS: demographic and health survey
aOR: adjusted odds ratio
cOR: crude odds ratio
CI: confidence interval
SR-STIs: self-reported sexually transmitted infections
WHO: World Health Organization
